# Analysis of Limiting Factors Across the Life Cycle of Delta Smelt (*Hypomesus transpacificus*)

**DOI:** 10.1007/s00267-018-1014-9

**Published:** 2018-05-09

**Authors:** Scott A. Hamilton, Dennis D. Murphy

**Affiliations:** 1 Hamilton Resource Economics, 7718 Davin Park Dr, Bakersfield, CA 93308 USA; 2 Department of Biology, University of Nevada, Reno, NV 89557 USA

**Keywords:** Limiting factors, Population ecology, Conceptual ecological models, Delta smelt, Sacramento-San Joaquin Delta

## Abstract

We developed a mechanistic life-cycle model derived from the elicitation of multiple factors influencing the success of individual life-stages of the imperiled delta smelt (*Hypomesus transpacificus*). We discuss the relevance of limiting factors in population ecology and problems with additive models in detecting them. We identify limiting factors and assess their significance using a non-linear optimization routine, combined with traditional metrics to assess the value of covariates and model performance. After reviewing previous conceptual models and multivariate analyses, we identified a set of factors that were consistent with conceptual models and useful in explaining the erratic fluctuations in a common abundance index: food at certain times in certain locations, predation by introduced species primarily in the spring, and entrainment. The analytical approach provides a transparent and intuitive framework in which to consider the contribution of covariates and consequences for population trends, and has the potential to assist with the evaluation of proposed recovery measures.

## Introduction

The concept of limiting factors is derived from the “theory of the minimum” developed by Carl Sprengel in 1839 and subsequently as Liebig’s Law of the Minimum. In its earliest applications it states that the yield of a crop is controlled not by the total amount of resources available, but by the availability of the scarcest resource—the limiting factor. The concept was derived from observations that applying more of a nutrient that was not limiting did not improve crop yields. The concept of limiting factors more recently has been applied to explain ecological phenomena (McNamara and Houston 1987; Klein 1991; Messier 1991; Thomson et al. 1996; Cade et al. 1999; Rettie and Messier 2000; Dunham et al. 2002; Kaiser et al. 1994). Recognition of the role of limiting factors in determining population responses in imperiled species is relevant for two reasons. First, it suggests that when certain limiting environmental factors control the distribution and abundance of a species, other factors are not doing so. A limiting-factors approach requires that only the data points for covariates that regulate the population of concern should be included in an analysis of environmental factors that affect the population, and conversely data points for covariates that are not limiting should be excluded from the analysis. Second, the manifestation of a limiting factor at some point in the future can nullify years of targeted resource management, since a contemporary limiting factor may serve to establish a new, lower level of the abundance of a species, eliminating previous gains. Identifying and managing to alleviate the effects of environmental factors that may limit the performance of a species in the future is important for sustaining the benefits of conservation actions already undertaken. Identifying and managing to reduce the deleterious effects of limiting factors on species of concern is prerequisite to successful conservation efforts.

We use the term “limiting factors” to refer to a set of factors that may potentially limit the abundance of a species or the size of a population. When one of those factors regulates abundance at a given time, hence is more limiting than the others, we refer to it as the “controlling factor.” We distinguish limiting factors (factors that may determine the maximum abundance of a given life stage, such as prior abundance, food availability, extent of habitat) from modifying factors (which reduce abundance from a level previously established by a controlling factor). Controlling factors can create bottlenecks wherein a large population may suddenly be reduced by a stressor that occurs seasonally or infrequently (Bisson PA (1989). Importance of the identification of limiting factors in an evaluation program. Unpublished manuscript. Available from P. Bisson, Weyerhaeuser Co). Controlling factors can confound the detection of population-dynamic thresholds or change points, such that small changes in an environmental driver may suddenly appear to produce large responses by a species of concern (Dodds et al. 2010). The abundance of a species at any point in time is a consequence of a previously manifested controlling factor and subsequent modifying factors. The presence and manifestation of limiting factors can be difficult to detect because they do not always control the response variable. When they do not control the observed response, those data points should not be given weight in a factor analysis (Kaiser et al. 1994; Cade et al. 1999); yet conventional additive approaches do just that (Augspurger 1996; Thomson et al. 1996, and see Appendix A).

Here, we present an approach to identifying the factors that limit populations only during certain seasons or stages, and apply the approach to explain variation and trend in a common abundance index for delta smelt (*Hypomesus transpacificus*) in the Sacramento-San Joaquin Delta in central California. Delta smelt are endemic to the upper San Francisco Estuary; the species was listed as threatened under the U.S. Endangered Species Act in 1993. Intensive study over more than 20 years has not resulted in the implementation of management measures that have reversed the species’ decline. The results of quantitative analyses of multiple environmental stressors on delta smelt have been inconsistent (MacNally et al. 2010; Thomson et al. 2010; Maunder and Deriso 2011; Miller et al. 2012; Rose et al. 2013a, b). As a consequence, an effective management strategy that could provide sustained benefits to delta smelt has not emerged and abundance index values for the species’ abundance are at historically low levels, less than 1% of that at the time of its listing.

We use a mechanistically defensible, conceptual ecological model that depicts the relations between environmental factors and the fish’s survival or reproduction during distinct life stages. The conceptual model provides the template for a stage-based life-cycle model that explores annual variation in the abundance of delta smelt and identifies the environmental factors that are associated with changes in abundance. We build life-stage-specific quantitative models by first articulating the hypothesized relationships between delta smelt and environmental factors as equations. We computerize the models, run analyses, and select a model by sequentially evaluating the value of covariates in explaining population responses. We consider the validity of the models on the basis of ecological plausibility and statistical strength. Our results may inform directed management actions that have potential to benefit delta smelt.

## Methods

**Fig. 1:**
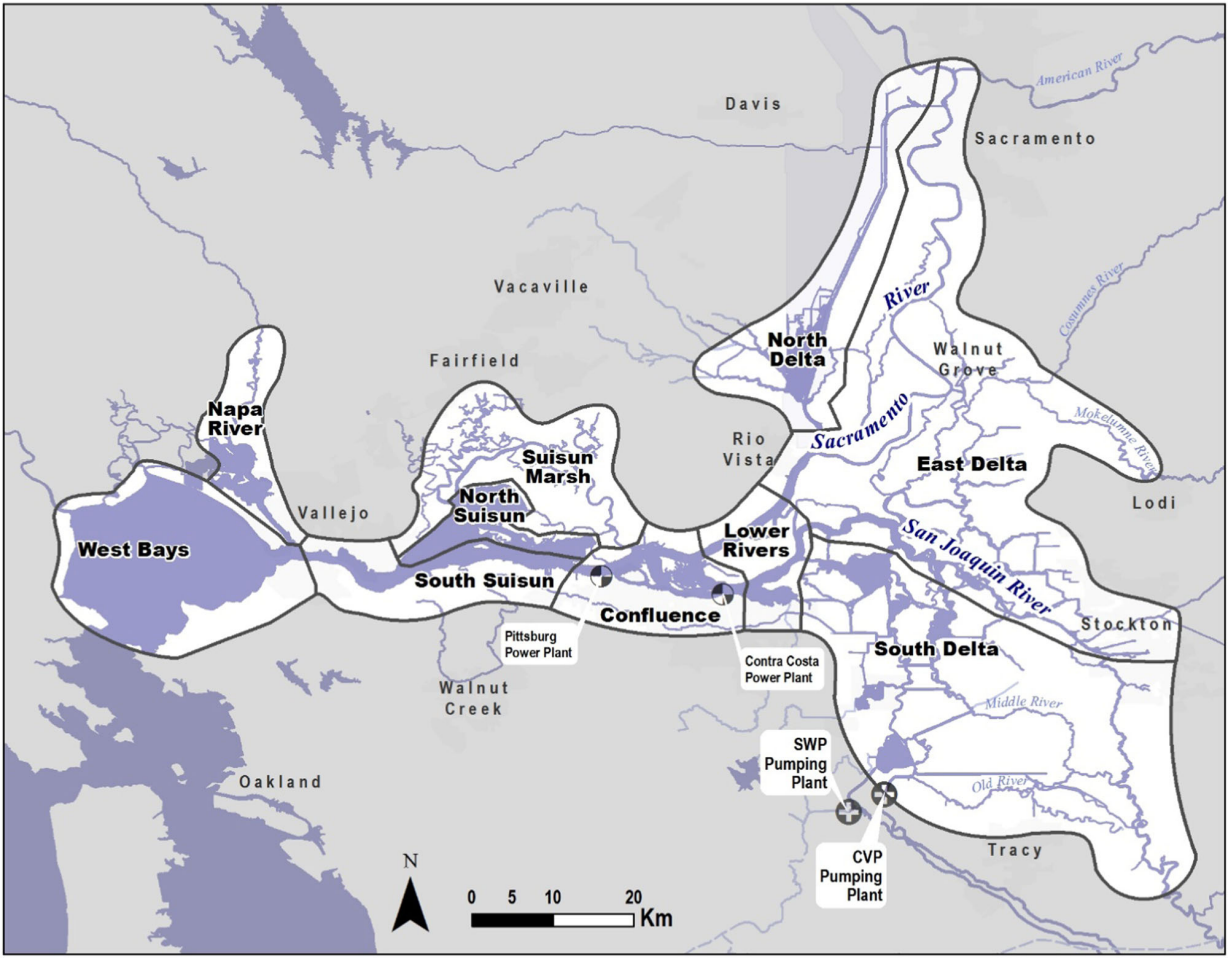
The San Francisco Estuary between San Francisco Bay and Sacramento, and regions used in this study

The San Francisco Estuary (Fig. 1), which supports the delta smelt, is among the most altered aquatic ecosystems in the United States (Nichols et al. 1986; Whipple et al. 2012; Cloern and Jassby 2012). More than 95% of the historic tidal wetlands in the Sacramento-San Joaquin Delta, including shallow freshwaters, riparian communities, and floodplains, have been lost to levees, hard channels, urbanization, and agriculture (Thompson 1957). Delta inflows during spring have been halved by upstream reservoirs and diversions (CDWR 2016). More than 200 non-native plant and animal species have become established and dominate many of the Delta’s ecological communities (URS 2007). Dozens of contaminants have accumulated in Delta substrates and continue to be delivered to its waters (Healey 2007). The densities of the delta smelt’s copepod prey are now at a fraction of that two decades ago (Kimmerer 2011). And introduced predators consume delta smelt as turbidity decreases due to sediment entrainment behind upstream dams, changes in land use, and invasive aquatic vegetation that attenuates currents (Jassby et al. 2002). Interactions among these and other environmental stressors have confounded analyses of the causes of the decline in delta smelt abundance (Sommer et al. 2007).

### Conceptual Ecological Model

**Fig. 2:**
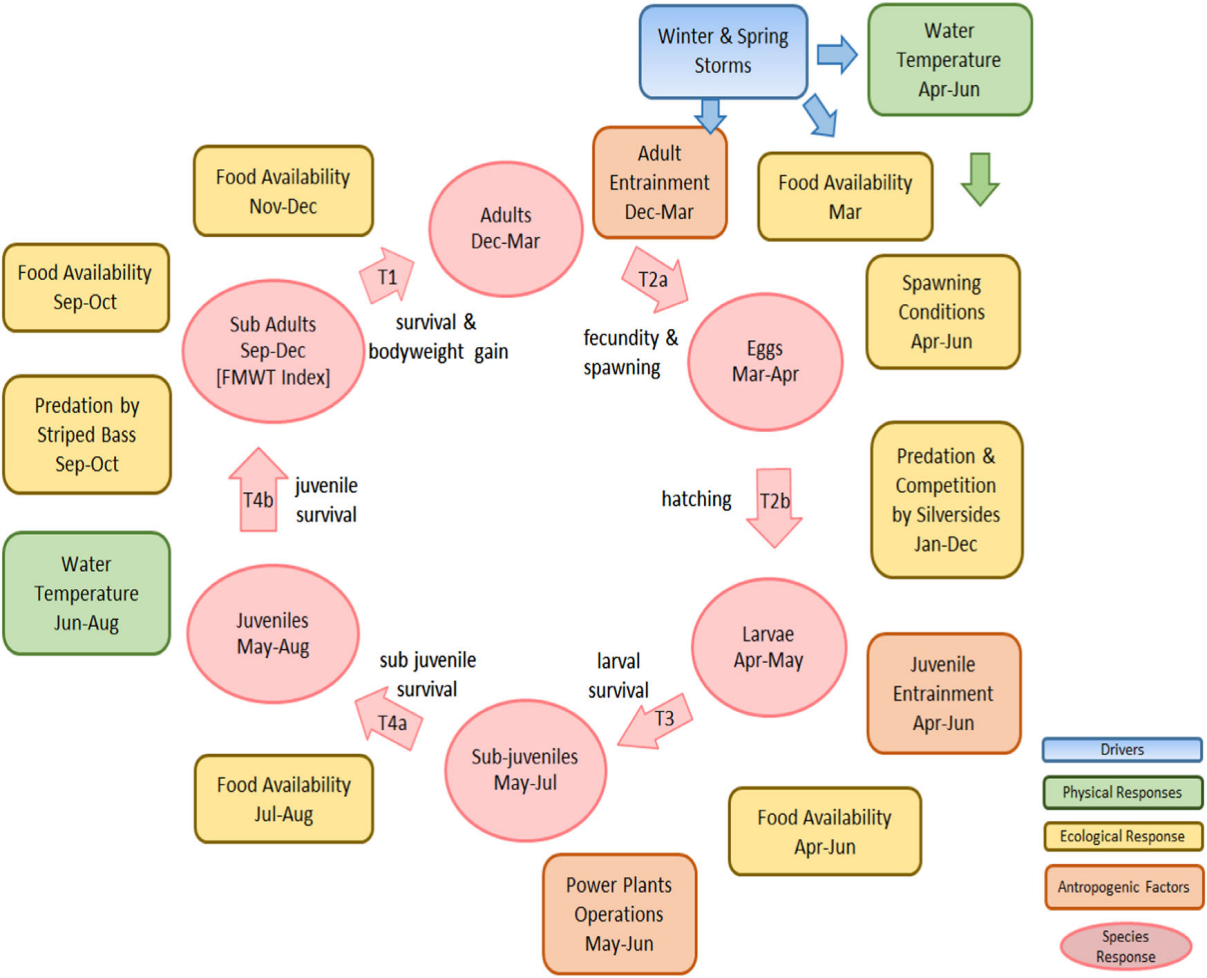
Conceptual ecological model of the life stages of delta smelt and the ecological influences on them

A foundation to conservation planning is the development of a conceptual ecological model. A number of conceptual models have been developed for delta smelt (Armor et al. 2005; Bennett 2005; Baxter et al. 2008; Miller et al. 2012; IEP MAST 2015; Moyle et al. 2016). These models identify a large number of environmental factors that plausibly may directly or indirectly affect the abundance of delta smelt; these factors vary in their responsiveness to management. Our conceptual ecological model for delta smelt (Fig. 2) draws from Moyle et al. (2016).

The primary drivers of delta smelt survival are winter and spring storms that vary in number and intensity among years, leading to profound differences in abiotic conditions, habitat availability and quality, and food-web dynamics. Delta smelt are affected by multiple additional environmental stressors, including predation on them and competition from non-native fishes and entrainment at pumps that export freshwater from the Delta.

Census data are not available for delta smelt; however, insight into the status of delta smelt can be derived through general fish surveys conducted throughout the year. The Fall Mid-water Trawl (FMWT) survey (http://www.dfg.ca.gov/delta/projects.asp?ProjectID=FMWT) has been conducted since 1967 and typically is implemented monthly from September through December. More than 180 stations (sites) have been sampled by the FMWT. Since 1991, 116 stations have been sampled in at least 20 years. Due to the large number of stations and the long period of data collection, we chose the abundance index developed from FMWT survey catch data (http://www.dfg.ca.gov/delta/projects.asp?ProjectID=FMWT) as a proxy measure of the species’ abundance. Our quantitative, deterministic, and mechanistic life-cycle model is designed to emulate the conceptual ecological model.

The translation of a conceptual model into useful quantitative models can be challenging. Rose (2000) pointed to six challenges of quantifying environmental effects on fish populations: detectability, complex and non-intuitive responses, a tendency to sacrifice realism when making regional predictions, interactions between communities, sub-lethal effects, and cumulative effects. We suggest that a seventh challenge is failure to identify and incorporate limiting factors. The abundance of a species is determined by the ability of its ecosystem to support it or not. The abiotic and biotic factors that define the species’ relation with its habitats vary among seasons and years. The concept of limiting factors, applied at a population level, suggests that the abundance of a species at any point in time is a consequence of a previous controlling factor. For example, predation in spring may limit the abundance of delta smelt in 1 year and the availability of zooplankton prey in summer in another. When predation is controlling, the availability of prey may not be. Yet most quantitative approaches include both the relevant and irrelevant data points in their analyses, thereby incorrectly characterizing the influence of environmental factors on abundance (see Appendix A). The challenge is to identify environmental factors that limit abundance of delta smelt from among the many potential factors, such as food supply, predation, weather extremes, and anthropogenic stressors.

### Candidate Covariates and Model Structure

We reviewed previous multivariate studies to harvest factors that could contribute to the population dynamics of delta smelt. Multivariate autoregressive models indicated substantial support for a relation between abundance of delta smelt and one of 19 covariates: summer water temperature (Mac Nally et al. 2010). A Bayesian change-point analysis found that two of 19 covariates, water clarity and the volume of water exported from the Delta in winter, were associated with an autumn abundance index of delta smelt (Thomson et al. 2010). A state-space life-cycle model suggested that the delta smelt abundance index is affected by density dependence, temperature from April through June and in July, prey density from April through June and July through August, abundance of predators from April through June and September through December, Secchi depth in January and February, and adult entrainment (Maunder and Deriso 2011). Five covariates met selection criteria employed by Miller et al. (2012): stock, entrainment, water temperature, prey densities, and predation from April through June. Rose et al. (2013a) developed a bioenergetics model that partitioned the Delta into 11 geographic regions. They assumed delta smelt dispersed in response to salinity, and they did not include predation in their model. They concluded that five factors affected abundance of delta smelt: salinity, water temperature (affecting the duration of the spawning window and bioenergetics of all life-stages), zooplankton, hydrodynamics (primarily via effects on entrainment), and number of eggs per spawning age-1 adult (primarily driven by the body weight of spawning adults).

Common among these studies (and consistent with the conceptual ecological model) are associations between the abundance of delta smelt and prior abundance, food in multiple seasons, water temperatures in spring (at the time of spawning) and summer (perhaps related to bio-energetic stress), turbidity, predation, and entrainment at water project facilities. Absent from all of the models summarized above, but consistent with earlier studies (Stevens and Miller 1983; Dege and Brown 2004; Nobriga et al. 2008; Kimmerer et al. 2009), was an association with freshwater flows into or through the Delta. Our conceptual model indicates that some functional flows influence food production, thus have an indirect, rather than a direct, effect on the abundance of delta smelt.

We explored whether the covariates in our conceptual ecological model were associated with each of four life stages. The first life-stage transition of delta smelt, from sub-adult in autumn to pre-spawning adult in winter (December through March), is a function of the abundance of sub-adults in autumn and the survival rate from autumn to pre-spawning. We hypothesized that the survival rate is limited by either food availability (Moyle et al. 1992; Lott 1998; Kimmerer and Orsi 1996; Nobriga 2002; Moyle 2002; IEP MAST 2015), or population potential after accounting for entrainment at water project facilities (Kimmerer 2008; Grimaldo et al. 2009; Miller 2011). Data on food availability in January and February are not consistently available, so we omitted this covariate. (The correlation of food availability in January and February with food availability in March from 1977 through 2014 was 0.82.) We estimated entrainment of adults as a function of salvage of adults at fish salvage facilities in the south Delta (IEP MAST 2015). We modeled the abundance of pre-spawning adults as1where *A*_m_ is the estimated abundance of mature (pre-spawning) adults in FMWT index units, *A*_f_ is the estimated FMWT index from the prior year, *E*_a_ is the salvage of adults at State Water Project (SWP) and Central Valley Project (CVP) pumping facilities from December through March, *M*_1_ is a constant (an estimated survival rate during the transition from sub-adults to pre-spawning adults), ɠ(*F*_ND_) is a function of the average prey availability (g/m^3^ carbon) in November and December in areas considered to be suitable for delta smelt, ɠ(*F*_M_) is the average prey availability (g/m^3^ carbon) in March, and *β*_1_ is an estimated coefficient. (See Eq. 5c for the form of the function ɠ).)

The second life stage transition, Eq. (2a), is that from maturity to spawning (April through June). The number of eggs produced is not measured or recorded because eggs are not observable in the wild. We hypothesized that the number of eggs is a function of the number of mature adults and the number of eggs per female (Rose et al. 2013a). The number of eggs per female is a function of the bodyweight of females and the average number of spawning events (Rose et al. 2013a). Because bodyweight of females is not recorded, we hypothesized that bodyweight of females at spawning is a function of food availability in autumn and winter (Rose et al. 2013b; IEP MAST 2015). The average number of spawning events is not recorded, so we hypothesized that it is a function of the duration of the spawning window. The duration of the spawning window in any year can be ascertained from the presence of larvae in the 20 mm survey. Although this survey does not accurately estimate the abundance of larvae because the gear was designed to catch larger fishes, it samples a large number of larvae (30% of the delta smelt surveyed are larval), which suggests it might provide a reasonable indication of presence, therefore the beginning and end of each spawning period. The 20 mm survey was initiated in 1995, and we sought data from 1975 onward to increase our sample size. We hypothesized that the duration of the spawning window is a function of the date at which temperatures become too warm for spawning, commonly thought to be 20 ^°^C (Bennett 2005; Rose et al. 2013a). We identified the date that the temperature of the Sacramento River at Rio Vista exceeded 20 ^°^C. We used data from Rio Vista because data collection began there in May 1983, the Sacramento River carries significantly more water into the Delta than other rivers, and because Rio Vista is approximately in the longitudinal center of the Delta, we hypothesized that water temperatures there might correlate with water temperatures in other regions of the Delta. However, time-series data for water temperatures at Rio Vista were too limited; therefore, we hypothesized that Rio Vista water temperature is a function of prior ambient air temperature (Wagner et al. 2011) and river flows at Rio Vista (Monismith et al. 2009).

Life-stage transition Eq. (2b) is the transition from egg to post-larva. The number of larvae is a function of the number of eggs and the survival rate from eggs to larvae. Survival from eggs to larvae has been hypothesized to be influenced by food availability (Nobriga 1998; Nobriga 2002; Hobbs et al. 2006; Slater and Baxter 2014), predation by and competition with silversides (*Menidia audens*) (Bennett and Moyle 1996; Bennett 2005; Baerwald et al. 2012; Mahardja et al. 2016), and early life-stage entrainment (Kimmerer 2008; Grimaldo et al. 2009; Miller 2011). Entrainment includes direct and indirect mortality associated with water diversions. Because mortality at the export pumps is not recorded, we hypothesized that water project-related entrainment is proportional to salvage at fish facilities (IEP MAST 2015). For entrainment related to generation of power at the Pittsburg and Contra Costa power plants, we hypothesized that entrainment was proportional to the power (MWH) produced in April and May with once through cooling technology. During April and May, delta smelt move back to the rivers confluence (Fig. 1) and adjacent areas following hatching, but are relatively poor swimmers and have difficulty avoiding the unscreened intakes (Matica and Sommer 2005). Thus, we modeled abundance of recruits (larvae) in any year as2awhere *A*_r_ is the estimated abundance of recruits, ƒ(*E*_j_) is a function of the expanded salvage of juvenile delta smelt at SWP and CVP pumping facilities from April through June, *M*_2_ is a constant (an estimated recruitment rate), *W* is the estimated spawning window in days (see Eq. (2b)), ƒ(*E*_p_) is a function of survival rate associated with the energy (MWH) produced at Pittsburg and Contra Costa power plants in May and June, ƒ(*P*_s_) is a function of the survival rate as a function of the abundance of silversides, which we estimated as the average catch per seine through the calendar year in beach seine surveys at the rivers confluence conducted by the US Fish and Wildlife Service, ?(*F*_S_) is a function of the average food availability in spring (April through June) in habitat (grams/m^3^ carbon), β is an estimated coefficient, and *A*_m_ is defined as above. (See Eq. 5a for the form of the function ƒ and Eq. 5c for the form of the function ɠ).)

We derived an estimate of the duration of the spawning window from water temperatures at Rio Vista using a quadratic function:

2b $$W = \beta _{0}+\beta _1T_{{\mathrm{A}}} + \beta _2T^2_{\,\,\, R}$$

where *W* is the spawning window in days calculated as the difference in days between the 5th percentile and the 95th percentile of larval delta smelt observed in the twenty millimeter survey, *T*_R_ is the Julian day at which water temperatures at Rio Vista Exceed 20 ^°^C and the *β* coefficients are calculated using ordinary least squares. The twenty-millimeter survey began in 1995. Therefore, the duration of the spawning window was estimated for all years using Eq. (2b). Data for *T*_R_ was not available prior to 1983 so estimates for *T*_R_ were derived from prior ambient air temperatures and river flow at Rio Vista:

2c $${{T}}_{\mathrm{R}} = \beta _{0} + \beta _1{{T}}_{{\mathrm{A}}} + \beta _2{{Q}}_{\mathrm{R}}$$

where *T*_A_ is the preceding ambient air temperature as determined by the prior 15-day average air temperature at Davis, *Q*_R_ is the average daily flow at Rio Vista (ft^3^/second) and the *β* coefficients are calculated using ordinary least squares.

The third transition is from post-larval to juvenile (July and August for modeling purposes). The number of juveniles is a function of the number of recruits and the survival rate of juveniles. We hypothesized that this survival rate is a function of food availability in suitable conditions (Nobriga 2002; Hobbs et al. 2006; Slater and Baxter 2014) and summer water temperatures (Mac Nally et al. 2010). Water temperature directly affects delta smelt metabolic rates, and, if sufficiently high, can induce stress and even mortality (IEP MAST 2015). Water temperatures primarily are influenced by air temperature, wind, tidal dispersion, and riverine flows (Monismith et al. 2009; Wagner et al. 2011). Thus, we modeled abundance of sub-juvenile delta smelt in any year as3where *A*_j_ is the estimated abundance of juveniles; ƒ(*T*_w_) is a function of the ambient summer temperature calculated from a transformation of the maximum 15-day average, ambient air temperature as measured at Davis, California, during June, July and August; *M*_3_ is a constant (an estimated survival rate), ɠ(*F*_JA)_ is a function of the average food availability in July and August in habitat (g/m^3^ carbon), and *A*_r_ is defined as above. (See Eq. 5a for the form of the function ƒ and Eq. 5c for the form of the function ɠ).)

The fourth transition is from sub-juveniles through juveniles to sub-adults (September through November). The number of sub-adults is a function of the number and survival rate of juveniles. We hypothesized that this survival rate is a function of food availability (Moyle et al. 1992; Lott 1998; Feyrer et al. 2003) and predation by striped bass (*Morone saxatilis*), which we in turn hypothesized is a function of striped bass abundance (Stevens 1963; Stevens 1966; Thomas 1967). Thus, we modeled abundance of sub-adult delta smelt in any year as4where *A*_e_ is the estimated FMWT Index, *A*_j_ is the estimated abundance of sub-juveniles, *M*_4_ is a constant (an estimated survival rate), ƒ(*P*_b_) is a function of the effect of predation by striped bass on survival (assumed to be a function of the abundance of striped bass in the September Fall Mid-water Trawl survey and the average Secchi depth in September and October (as measured by the FMWT survey and calculated at stations from Suisun Bay to the Lower Sacramento River), ɠ(*F*_SO_) is average food availability in September and October in habitat (g/m^3^ carbon). (See Eq. 5a for the form of the function ƒ and Eq. 5c for the form of the function ɠ).)

We estimated predation, ambient temperature and entrainment at power plants ƒ(*P*_s_) ƒ(*P*_b_) ƒ(*E*_p_) as logistic functions:

5a $$\mathrm{f}\left( {{X}} \right) = \left( {e^{\left( {{\mathrm{\beta }}_0 + {\mathrm{\beta }}_1{{X}}} \right)}} \right){\mathrm{/}}\left( {1 + e^{\left( {{\mathrm{\beta }}_2 + {\mathrm{\beta }}_1{{X}}} \right)}} \right) + {\mathrm{\beta }}_3$$

where *β* is an estimated coefficient. This functional form is a curve with a survival rate of 1 at low levels of *X*, decreasing to a survival rate of *β*_3_ at high levels of *X*.

To reduce the number of parameters estimated, we calculated, rather than estimated, an approximation for *β*_2_ as

5b $${\mathrm{\beta }}_2\, = {\mathrm{\beta }}_{0} + {\mathrm{\beta }}_{3} + {\mathrm{\beta }}_3^{\mathrm{e}}$$

We did not consider density dependence as a factor limiting the abundance of delta smelt per se (see Maunder and Deriso 2011; Miller et al. 2012), but rather considered factors that might lead to density dependence. We used exponential rather than multiplicative coefficients for food and the duration of the spawning window to avoid generating a non-unique estimate of a coefficient.5c

### Data Sources and Specification of Covariates

We compared the covariates included in our models (Eqs. 1–5) (Table 1) with covariates that were strongly associated with delta smelt abundance in prior analyses. They include covariates related to prior abundance (Miller et al. 2012); number of eggs per spawning adult (Rose et al. 2013a); spring water temperatures (Maunder and Deriso 2011; Miller et al. 2012; Rose et al. 2013a), which we hypothesized affect the duration of the spawning window; entrainment (Miller et al. 2012; Rose et al. 2013a); spring predation (Maunder and Deriso 2011; Miller et al. 2012); autumn predation (Maunder and Deriso 2011); food availability at multiple life-stages (Maunder and Deriso 2011; Miller et al. 2012; Rose et al. 2013a); summer water temperatures (Maunder and Deriso 2011; Mac Nally et al. 2010; Rose et al. 2013a); water clarity (Thomson et al. 2010; Maunder and Deriso 2011), which we hypothesized affects both habitat quality for delta smelt and predation by striped bass; and salinity (Rose et al. 2013a), which we hypothesized affects habitat quality for delta smelt. We did not include winter exports (Thomson et al. 2010), although we included adult salvage and hydrodynamics; hydrodynamics primarily may affect entrainment (Rose et al. 2013a). Consequently, although we developed a covariate set by considering first principles, we believe we have given appropriate consideration to covariates that were found relevant in prior multivariate analyses. Also, we do not make any a priori assumptions regarding density dependence (see Maunder and Deriso 2011); rather, we expect that the density of delta smelt is mediated by the availability of food.

**Table 1:** Candidate covariates included in the analysis of factors influencing the abundance of delta smelt

Factor by life-stages	Abbreviated name	Metric	Source data
Sub-adult to spawning adults			
Prior FMWT index	Stock	FMWT index	FMWT^f^
Food availability: Nov–Dec, Mar	Nov–Dec Food Mar Food	Biomass of adult calanoid copepods^a^	Zooplankton^h^
Adult entrainment	Adult Entr	Salvage of adult delta smelt Dec–Mar	Salvage^g^
Spawning adults to larvae			
Estimated duration of the spawning window	Spawn Window	Estimated duration of the spawning window^b^	20 mm^i^
			CDEC^j^
Food availability for larvae: Apr-Jun	Apr–Jun food	Biomass of calanoid copepodids and adults, and cyclopoid adults^c^	Zooplankton^h^
Abundance of silversides	Silversides	Average catch of silversides in the Confluence	Beach Seine^k^
Entrainment at water projects	Juv Entr	Salvage of juvenile delta smelt Apr–Jun	Salvage^g^
Juveniles			
Food availability: Jul–Aug	Jul–Aug food	Biomass of adult calanoid copepods^a^	Zooplankton^h^
Summer temperature	Summer Temp	Mean ambient temperature Jun–Aug^d^	UCD^m^
Power plant operations	Power plants	Combined power generation at Contra Costa and Pittsburg power plants May–Jun	US EIA^l^
Sub-adults			
Food availability: Sep–Oct	Sep–Oct food	Biomass of adult calanoid copepods^a^	Zooplankton^h^
Abundance of striped bass	Striped Bass	Abundance of striped bass in September^e^	FMWT^f^

^a^Grams of carbon in a cubic meter of water sampled in the zooplankton survey (see (h)) at stations with water samples values between the lower and upper ranges of abiotic attributes specified in Table 2 based on the density (and g of carbon) in the following species *Acartiella sinensis* (3 g), *Diaptomidae* (3 g), *Eurytemora affinis* (2.5 g), *Pseudodiaptomus forbesi* (3 g), *Pseudodiaptomus marinus* (5 g), *Sinocalanus doerrii* (4 g), *Tortanus* spp. (5.4 g), and other calanoid adults (3 g). *Acartia* spp. were excluded as they primarily occur in higher-salinity waters

^b^Derived from the estimated first Julian date that water temperatures at Rio Vista exceed 20 ^°^C. The equations are *W* = −0.0294T_R_^2^ + 9.27T_R_ − 657.2 where *R*^2^ = 0.763, *n* = 19, all *P*-values less than 0.001 *T*_R_ = 8.878 + 0.767T_A_−0.524Ln(Q_R_) where *R*^2^ = 0.951, *n* = 10,397, all *P*-values less than 0.001

^c^Calculated by adding to (a) the grams of carbon contributed by copepodids of the same species listed in (a) assigning 1 g of carbon to each, and carbon weights of adult cyclopoids assigning to each: *Acanthocyclops vernalis* (3 g), *Limnoithona* spp. (0.3 g), *Limnoithona sinensis* (0.3 g), *Limnoithona tetraspina* (0.3), *Oithona davisae* (0.2 g), *Oithona similis* (0.5), *Oithona* spp. (1.0)

^d^Maximum of the 15 day average air temperature in June through August at University of California, Davis (see[m]).

^e^Striped bass catch in September in the Fall Mid-water Trawl (FMWT) survey in North and South Suisun, Confluence and Lower Rivers. See (f)

^f^CDFW Fall Mid-water Trawl (FMWT) Survey ftp://ftp.dfg.ca.gov/YoungFishesProject/FMWT%20Data/

^g^CDWR Salvage data ftp://ftp.dfg.ca.gov/salvage

^h^CDFW Zooplankton Survey by request from DFW at http://www.water.ca.gov/bdma/meta/zooplankton.cfm

^i^CDFW 20MM Survey ftp://ftp.dfg.ca.gov/Delta%20Smelt/20-mm.mdb

^j^CDEC http://cdec.water.ca.gov/cgi-progs Rio Vista (D24A)

^k^USFWS Beach Seine Survey http://www.fws.gov/lodi/jfmp

^l^US Energy Information Association https://www.eia.gov/opendata/qb.cfm?category=1154&sdid=ELEC.PLANT.GEN.228-ALL-ALL.M

https://www.eia.gov/opendata/qb.cfm?category=1189&sdid=ELEC.PLANT.GEN.271-ALL-ALL.M

^m^University of California, Davis average air temperature

We subdivided the upper estuary into ten regions, seven of which had sufficient historic data on the covariates of interest (Fig. 1).

Given that analyses of the gut contents of delta smelt have shown that food type varies throughout the year (IEP MAST 2015), there are multiple ways of specifying prey availability. For the sake of parsimony, we considered food availability only in areas that we considered had suitable abiotic conditions for delta smelt. For all seasons except spring (April–June), we multiplied the average biomass of adult calanoid copepods for each region and month, or groups of months, by the proportion of stations considered to have suitable abiotic conditions for delta smelt. For example, if the south Delta had high densities of food, but the water in summer was too clear for delta smelt at all stations in the region, food availability in that region would be zero. Rather than develop an arbitrary definition of suitable abiotic conditions, we assessed abundance in the spring Kodiak, twenty millimeter, summer tow net, and fall mid-water trawl surveys. For each survey, we calculated the temperature, salinity, and turbidity (Secchi depth) associated with the 5 and 95% percentile of delta smelt in survey samples (Table 2). Stations where all three abiotic parameters were within those percentiles were considered to have suitable abiotic conditions.

**Table 2:** Abiotic delineations associated with the 5th and 95th percentile of delta smelt abundance in each survey

Months	Source	Period	Temperature (^o^C)		Secchi depth (cm)		Salinity (μs/cm)	
			Lower	Upper	Lower	Upper	Lower	Upper
Jan–Mar	SKT	2002–2014	7.9	18.2	10	75	155	8678
Apr–Jun	20 mm	1995–2014	10.0	22.3	10	87	100	7720
Jul–Aug	STN	1973–2014	10.0	23.6	10	56	143	16,482
Sep–Dec	FMWT	1967–2014	7.8	20.8	10	74	161	15,698

*Sources*: SKT—Spring Kodiak Trawl; 20–20 mm Trawl; STN—summer tow-net; FMWT—Fall mid-water Trawl. See notes under Table 1 for source data

For food availability during the spring, we included adult calanoid copepods, calanoid copepodids, and cyclopoid adults as potential prey for larval delta smelt in April and May, which might seek relatively small copepods. We considered food availability for larvae in three regions: Montezuma Slough, the Confluence and lower rivers, and Suisun March. In 60% of years, more than 50% of delta smelt were observed in Montezuma Slough. Abundance of delta smelt in May and June is greatest in the Confluence and lower rivers. Suisun Marsh might represent other areas where delta smelt spawn, but there are no data from that region.

We checked for covariates that were highly correlated for with other covariates (*R* > 0.5).

### Statistical Methods

Mathematical programming can be used to conduct regression analysis (Wagner 1958; Wang et al. 2004). The objective function is to select a set of coefficients, such that the difference between the observed and predicted value of the response variable is minimized, by minimizing the residual sum of squares. We used a generalized reduced-gradient non-linear optimization routine to minimize the residual sum of squares between the predicted and observed annual FMWT Indexes over 39 years (1975–2014; a full FMWT survey was not conducted in 1979). We incorporate prior abundance (the abundance-index value of the population at the start of each life-stage) and food availability as factors that may be potentially limiting.

Those limiting factors may be subsequently affected by modifying factors. The analysis selects the minimum (the most limiting) of these factors during each life stage. All of the factors not contributing to the minimum are excluded from the analysis in that life stage and generation.

Our model calculates an abundance index potential in each of four life stages on the basis of the abundance index at the start of each life stage and modifying factors. Separately, the model calculates the population size that may be supported by the available food. The routine then selects the minimum of the two. In this formulation, each year contains equations for the four life-stage transitions and provides one annual estimate of autumn abundance index with one residual.

The relevance of the available data is determined by considering changes in the Akaike Information Criterion adjusted for small sample sizes (AICc) (Burnham and Anderson 2002).

The incorporation of limiting factors into an analysis can create demands on available degrees of freedom, because each limiting factor, when controlling, subdivides that data set. For example, suppose:6a

In any given year either prior abundance [*A*_f_ *M*] or food, ɠ (*F*) will be controlling. Thus, two sets of equations are being estimated:

6b $${{A}}_{\mathrm{m}} = {{A}}_{\mathrm{f}}{{M}}$$

6c

We sought to identify a set of models for which there was substantial support, with AICc differences less than two, following Burnham and Anderson (2002) and strong explanatory power, with *R*^2^ greater than 0.7), by considering the explanatory value that a covariate provides to the aggregated model (Eqs. (1–4), where all coefficients are estimated simultaneously). To avoid problems of over specification in early model runs, the initial model included only the limiting factors (the prior abundance-index value, and food availability) and one multiplier (*M*_2_). We then ran the initial model without each of the limiting factors. If AICc was reduced when a factor was excluded, the covariate was removed from the model. Next, we added all the modifying factors, and then sequentially eliminated variables the exclusion of which provided the largest reduction in AICc. If the model with the minimum AICc had coefficients with plausible signs and in credible ranges, we then repeated the process of removing a covariate until the AICc no longer could be reduced. Also, to ensure the above procedure for selecting variables did not exclude relevant variables or include irrelevant variables, we added each excluded variable into the model with the lowest AICc value and excluded each included variable, and then compared AICc statistics among models, including AICc differences.

We calculated the AICc value (following Burnham and Anderson 2002):

7a $${\mathrm{AICc}} = - 2 \ast {{L}} + 2{{K}} + 2{{K}}({{K}} + 1){\mathrm{/}}\left( {{{n}} - {{K}} - 1} \right)$$

where *K* is the number of parameters, *n* is the number of observations, and *L* is the log-likelihood, which was calculated as:

7b $${{L}} = {{n/}}2 \ast {\mathrm{ln}}\left( {\sigma ^2} \right) - {{n/}}2({\mathrm{ln}}\left( {2\pi } \right) + 1)$$

where *σ*^2^ is the residual sum of squares divided by the number of observations; in this case the sum of the difference between the predicted and observed FMWT abundance-index value squared, divided by the number of years of available data.

We applied the information-theoretic approach to identify the covariates most strongly associated with the response variable by considering Akaike weights (*W*_i_)–a measure of the relative closeness of a particular model to the best model in the set. We calculated *W*_i_ for model *i* are calculated as

8 $$w_{i = }(e^{ - \Delta _{i/2}}){\mathrm{/}}\mathop {\sum}\limits_{{{r}} = 1}^{{R}} {e^{ - \Delta _{r/2}}}$$

where Δ_i_ = AICc_i_−AICc_min_ and *R* is the number of models in the set.

For each model, we recorded the bias-adjusted Akaike’s information criterion (AICc), the simple differences in AICc from the model with the minimum AICc (Δ_i_), the Akaike weights (*W*_i_), and *R*^2^, and examined the plots of residuals vs. fitted values for evidence of unequal variances, and then inspected plots of residuals vs. covariates for evidence of a pattern that might indicate model misspecification or the need for a transformation of the independent variable (Neter et al. 1996).

We estimated the relative support for covariates *x*_j_ by calculating *w*_+_(*j*), the sum of Akaike weights across all the models in the set where the variable *j* occurs. The relative strength of support for variable *j* is reflected in the total *w*_+_(*j*). The larger the *w*_+_(*j*), the more strongly variable *j* is supported relative to the other covariates.

### Model Validation

We considered the validity of the selected model in six ways. First, we conducted a cross-validation by leaving out one annual observation (one delta smelt FMWT index value), then used the remaining data set to estimate new model coefficients and to predict the FMWT index for the omitted year. We repeated this process 39 times to obtained predictions for every year.

Second, we checked for adequate degrees of freedom. Partitioning the data into two subsets reduces the degrees of freedom and increases the risk of over-specification—that is, having too many coefficients for the available observations. As our rule of thumb, we desired to have degrees of freedom that were double the number of coefficients being estimated.

Third, we considered other data to assess the plausibility of the results.

Fourth, we considered the plausibility of the shape of the response functions.

Fifth, we correlated our predicted delta smelt seasonal abundances with survey returns from the Spring Kodiak Trawl (average February CPUE), the 20 mm Survey (average June CPUE), and the Summer Tow-net Survey (average July CPUE). Each of these surveys was designed to sample delta smelt or captured delta smelt as by-catch, although the temporal periods of the surveys do not correspond precisely with our life-stage transition intervals.

Sixth, we evaluated whether model results were consistent with the survey data. If delta smelt abundance in one season is a good predictor of abundance in the next season, environmental factors likely have effect on population size. When the variation is great it indicates that environmental factors have comparatively greater influence in regulating the population during that period. Comparing the timing of the influence of factors in the model with variability in abundance-index values between surveys provides an additional check of the plausibility of the factors in the model.

The results of the above model validation exercises are provided as supplementary material (Appendix B). A comprehensive description of the methods employed in this study is provided as supplementary material (Appendix C).

## Results

Correlations among covariates greater than 0.5 were few (see Table 3). Food availability in July and August was correlated with food availability later in the year reflecting an expected autocorrelation; food availability is partly a function of food availability in the previous period. That observation contributes to minimizing the number of food covariates that need to be considered to identify the most critical period (See Table 4). Abundance of silversides was negatively correlated with striped bass abundance, power-plant operations, and food availability in July and August. Adult salvage was correlated with power plant generation. These covariate correlations likely reflect trends over time and are likely not causally related. Adult and juvenile salvage were correlated with prior abundance—the more delta smelt in the estuary, the more they are likely to be entrained. Striped bass abundance was correlated with food availability in summer, an observation that should be considered in reference to the systematic pelagic organism decline in the estuary (see Armor et al. 2005). All other correlations were less than 0.5. We did not remove correlated variables from the analyses, but note that inclusion of correlated covariates in preferred models requires caution in their interpretation.

**Table 3:** Correlations(R) between covariates included in the analysis of factors influencing the abundance of delta smelt

	Stock	Silver-sides	Striped Bass	Adult Entr	Juv Entr	Power Plants	Summ Temp	Spawn Window	Food availability			
									Mar	Apr–Jun	Jul–Aug	Sep–Oct
Silversides	−0.42											
Striped Bass	0.03	−0.49										
Adult Entr	0.63	−0.43	0.19									
Juv Entr	0.70	−0.34	−0.04	0.69								
Power plants	0.45	−0.65	0.28	0.70	0.36							
Summ Temp	0.05	0.08	−0.19	0.09	0.15	−0.07						
Spawn Window	0.09	−0.12	0.20	0.18	0.14	0.02	−0.07					
Food availability												
Mar	0.12	−0.22	0.24	0.39	0.08	0.41	−0.08	0.06				
Apr–Jun	0.09	−0.08	0.28	0.00	0.02	−0.06	−0.27	−0.05	0.25			
Jul–Aug	−0.04	−0.56	0.50	0.12	−0.02	0.20	−0.17	0.25	0.10	0.21		
Sep–Oct	0.16	−0.33	0.18	0.00	0.02	−0.04	−0.07	0.23	−0.08	0.13	0.66	
Nov–Dec	0.03	−0.30	0.57	0.13	−0.04	0.25	0.04	0.12	0.18	0.22	0.57	0.18

See Table 1 for a key to abbreviations of covariate names

**Table 4:** Results for models with descending numbers of limiting factors

Model	Covariates included	df	AICc	Δ_i_	*W*_i_	*R* ^2^
1	Stock, Nov–Dec, Mar, Apr–Jun, Jul–Aug, Sep–Oct	23	593.0	42.0	0.0000	0.62
2	Stock, Mar, Apr–Jun, Jul–Aug, Sep–Oct	26	575.1	24.1	0.0000	0.62
3	Stock, Nov–Dec, Apr–Jun, Jul–Aug, Sep–Oct	26	575.1	24.1	0.0000	0.62
4	Stock, Nov–Dec, Mar, Jul–Aug, Sep-Oct	26	586.7	35.7	0.0000	0.49
5	Stock, Nov–Dec, Mar, Apr–Jun, Sep–Oct	26	582.8	31.8	0.0000	0.54
6	Stock, Nov–Dec, Mar, Apr–Jun, Jul–Aug	26	575.1	24.1	0.0000	0.62
7	Stock, Apr–Jun, Jul–Aug	32	551.0	0.0	0.5794	0.62
8	Stock, Apr–Jun	35	564.7	13.7	0.0006	0.31
9	Stock, Jul–Aug	35	551.6	0.6	0.4199	0.51
10	Stock	38	571.3	20.3	0.0000	0.01

*Note*: df–degrees of freedom, AICc–bias adjusted Akaike’s information criterion, Δ_i_ is–simple difference in AICc from the model with the minimum AICc, W_i_–the Akaike weight, *R*^2^–the coefficient of determination. See Table 1 for a key to abbreviations of covariate names. Model 1 includes all limiting factors. Models 2 through 6 have one factor eliminated. Food availability in November–December, March and September–October provided the least value and these factors were eliminated in model 7. Models 7 and 9 could not be distinguished on the basis of our selection criteria: Δ_i_ < 2. Models without stock (prior abundance in the fall) were not ecologically plausible

Equations [2b] and [2c] were estimated respectively as:

$${{W}} = 657.2 + 9.27\,{{T}}_{\mathrm{R}}-0.0294\,T^2_{\,\,\, R}\quad \quad R^2 = 0.76$$

$${{T}}_{\mathrm{R}} = 8.878 + 0.767\,{{T}}_{\mathrm{A}}-5.24\,{{Q}}_{\mathrm{R}}\quad \quad {{R}}^2 = 0.95$$

and were employed to produce the covariate *W*, the estimated duration of the spawning window for the entire period of the study.

### Model Selection

Recognizing that models with multiple limiting factors can readily over-fit data sets, we sought initially to reduce the number of limiting factors in the analysis from six food-availability and prior-abundance factors in Eqs. (1–4) by utilizing the AICc statistic to eliminate those factors that provided the least information. Models 7 and 9 (Table 4) had similar AICc values with no other models having AICc differences less than 10. Those models indicated that food availability in April through June and July through August were more strongly associated with abundance of delta smelt than food availability at other times of the year. We selected model 9 for further development on the basis that a model with one fewer covariate would reduce the chance of over-fitting when incorporating modifying factors. The validity of the selection is reviewed below.

We then added all the modifying factors, and sequentially eliminated the one factor contributing the least information, until the AICc statistic could be reduced no further (Model 30, Table 5). The resulting model included four factors: prior abundance, abundance of silversides, food availability in summer (July–August), and entrainment at power plants, and a recruitment parameter, M_2_ (*R*^2^ = 0.88). The inclusion of survival parameters (M_1_, M_3_, and M_4_) did not improve the AICc value.

**Table 5:** Results for models with descending numbers of modifying factors

Model	Covariates Included	df	AICc	Δ_i_	*W*_i_	*R* ^2^
11	Stock, Jul–Aug Food, Adult Entr, Spawn Window, Silversides, Juv Entr, Power Plants, Summer Temp, Striped Bass	20	565.4	48.6	0.0000	0.90
12	Stock, Jul–Aug Food, Spawn Window, Silversides, Juv Entr, Power Plants, Summer Temp, Striped Bass	21	557.1	40.3	0.0000	0.90
13	Stock, Jul–Aug Food, Adult Entr, Silversides, Juv Entr, Power Plants, Summer Temp, Striped Bass	21	556.7	39.9	0.0000	0.90
14	Stock, Jul–Aug Food, Adult Entr, Spawn Window, Juv Entr, Power Plants, Summer Temp, Striped Bass	23	561.1	44.3	0.0000	0.83
15	Stock, Jul–Aug Food, Adult Entr, Spawn Window, Silversides, Power Plants, Summer Temp, Striped Bass	21	556.5	39.7	0.0000	0.90
16	Stock, Jul–Aug Food, Adult Entr, Spawn Window, Silversides, Juv Entr, Summer Temp, Striped Bass	23	589.6	72.8	0.0000	0.65
17	Stock, Jul–Aug Food, Adult Entr, Spawn Window, Silversides, Juv Entr, Power Plants, Striped Bass	23	543.4	26.6	0.0000	0.89
18	Stock, Jul–Aug Food, Adult Entr, Spawn Window, Silversides, Juv Entr, Power Plants, Summer Temp	23	547.6	30.8	0.0000	0.88
19	Stock, Jul–Aug Food, Spawn Window, Silversides, Juv Entr, Power Plants, Striped Bass	24	535.9	19.1	0.0001	0.90
20	Stock, Jul–Aug Food, Adult Entr, Silversides, Juv Entr, Power Plants, Striped Bass	24	536.7	19.9	0.0000	0.89
21	Stock, Jul–Aug Food, Adult Entr, Spawn Window, Juv Entr, Power Plants, Striped Bass	26	543.7	26.9	0.0000	0.83
22	Stock, Jul–Aug Food, Adult Entr, Spawn Window, Silversides, Power Plants, Striped Bass	24	535.0	18.2	0.0001	0.90
23	Stock, Jul–Aug Food, Adult Entr, Spawn Window, Silversides, Juv Entr, Striped Bass	26	576.7	59.9	0.0000	0.60
24	Stock, Jul–Aug Food, Adult Entr, Spawn Window, Silversides, Juv Entr, Power Plants	26	529.8	13.0	0.0012	0.88
25	Stock, Jul–Aug Food, Spawn Window, Silversides, Juv Entr, Power Plants	27	524.9	8.1	0.0142	0.88
26	Stock, Jul–Aug Food, Adult Entr, Silversides, Juv Entr, Power Plants	27	524.9	8.1	0.0143	0.88
27	Stock, Jul–Aug Food, Adult Entr, Spawn Window, Juv Entr, Power Plants	29	531.0	14.2	0.0007	0.83
28	Stock, Jul–Aug Food, Adult Entr, Spawn Window, Silversides, Power Plants	27	524.9	8.2	0.0138	0.88
29	Stock, Jul–Aug Food, Adult Entr, Spawn Window, Silversides, Juv Entr	29	577.4	60.6	0.0000	0.43
30	Stock, Jul-Aug Food, Silversides, Power Plants	29	516.8	0.0	0.8121	0.88
31	Stock, Jul–Aug Food, Power Plants	32	520.3	3.5	0.1435	0.82
32	Stock, Jul–Aug Food, Silversides	32	566.5	49.7	0.0000	0.43
33	Stock, Jul–Aug Food, Silversides, Power Plants, Apr–-Jun Food	26	529.4	12.6	0.0015	0.88

*Note*: df–degrees of freedom, AICc–bias adjusted Akaike’s information criterion, Δ_i_–simple difference in AICc from the model with the minimum AICc, W_i_–Akaike weight, *R*^2^–coefficient of determination. See Table 1 for a key to abbreviations of covariate names. Model 11 includes the limiting factors from model 9 and all modifying factors. Covariates not included in the model with the lowest AICc value in each iteration were excluded from the next iteration. Model 30 had the lowest AICc value and Δ_i_ > 2 for all other models

As a check of our selection procedure, we inserted each excluded covariate into the model with the lowest AICc value, and removed each included covariate (Table 6), but no superior models emerged. A model with food availability in March had the closest AICc value with a Δ_i_ of 2.9. The Akaike weights for the covariates in the model with the lowest AICc value were greater than 0.92 (Table 7). No other covariates had weights exceeding 0.5. As a further check on the selection procedure, we considered additions of covariates to and deletions from to the next best model, the model (which included food availability in March) with the second lowest AICc value from Table 6. Inserting covariates in or removing covariates from this model did not lower the AICc. A model including juvenile entrainment had the closest AICc value. Numerous models in Table 6 had lower AICc values.

**Table 6:** Comparison of the model with the lowest AICc value (model 30) with model variants

Covariates	*K*	AICc	Δ_i_	*W*_i_	*R* ^2^	Adj *R*^2^
Covariates inserted						
Food availability (Sep–Oct)	14	530.5	13.7	0.0004	0.879	0.823
Summer temperature (Jun–Aug)	14	530.5	13.7	0.0005	0.879	0.824
Food availability (Apr–Jun)	14	529.4	12.6	0.0008	0.882	0.828
Abundance of striped bass	14	528.7	11.9	0.0011	0.885	0.832
Food availability (Nov–Dec)	14	523.0	6.2	0.0189	0.900	0.854
Juvenile survival (parameter)	12	521.0	4.2	0.0526	0.879	0.836
Sub-adult survival (parameter)	12	521.0	4.2	0.0518	0.879	0.836
Juvenile entrainment loss (Apr–Jun)	12	521.0	4.2	0.0522	0.879	0.836
Estimated duration of spawning window	12	520.3	3.5	0.0734	0.881	0.839
Adult survival (parameter)	12	520.3	3.5	0.0725	0.881	0.839
Adult entrainment (Dec–Mar)	12	520.3	3.5	0.0727	0.881	0.839
Food availability (Mar)	14	519.7	2.9	0.1011	0.908	0.866
Model with lowest AICc value	11	516.8	0.0	0.4266	0.879	0.842
Covariates removed						
Abundance of silversides	8	520.3	3.5	0.0754	0.825	0.792
Recruitment (parameter)	10	544.7	27.9	0.0000	0.726	0.653
Power plant operations (May–Jun)	8	566.5	49.7	0.0000	0.427	0.320
Prior fall mid-water trawl index	11	575.9	59.1	0.0000	0.450	0.279
Food availability (Jul–Aug)	8	596.3	79.5	0.0000	0.072	−0.217

*Note*: K–number of parameters, AICc–bias adjusted Akaike’s information criterion, Δ_i_ is–simple difference in AICc from the model with the minimum AICc, W_i_–Akaike weight, *R*^2^–coefficient of determination, Adj *R*^2^–the coefficient of determination adjusted for the number of parameters estimated. The top portion of the table depicts impacts on model performance if an individual covariate is added to the model with the lowest AICc value. The bottom portion of the table depicts impacts on model performance if an individual covariate is removed from the model with the lowest AICc value.

**Table 7:** Akaike weights of covariates generated from the models in Table 6

Covariate	*w*+(*j*)
Food availability (Mar)	0.3969
Estimated duration of spawning window	0.4247
Adult entrainment (Dec–Mar)	0.4253
Adult survival (parameter)	0.4255
Juvenile survival (parameter)	0.4454
Juvenile entrainment loss (Apr–Jun)	0.4458
Sub-adult survival (parameter)	0.4463
Food availability (Nov–Dec)	0.4791
Abundance of striped bass	0.4969
Food availability (Apr–Jun)	0.4973
Summer temperature (Jun–Aug)	0.4976
Food availability (Sep–Oct)	0.4976
Abundance of silversides	0.9246
Recruitment (parameter)	1.0000
Power plant operations (May–Jun)	1.0000
Prior fall mid-water trawl index	1.0000
Food availability (Jul–Aug)	1.0000

**Fig. 3:**
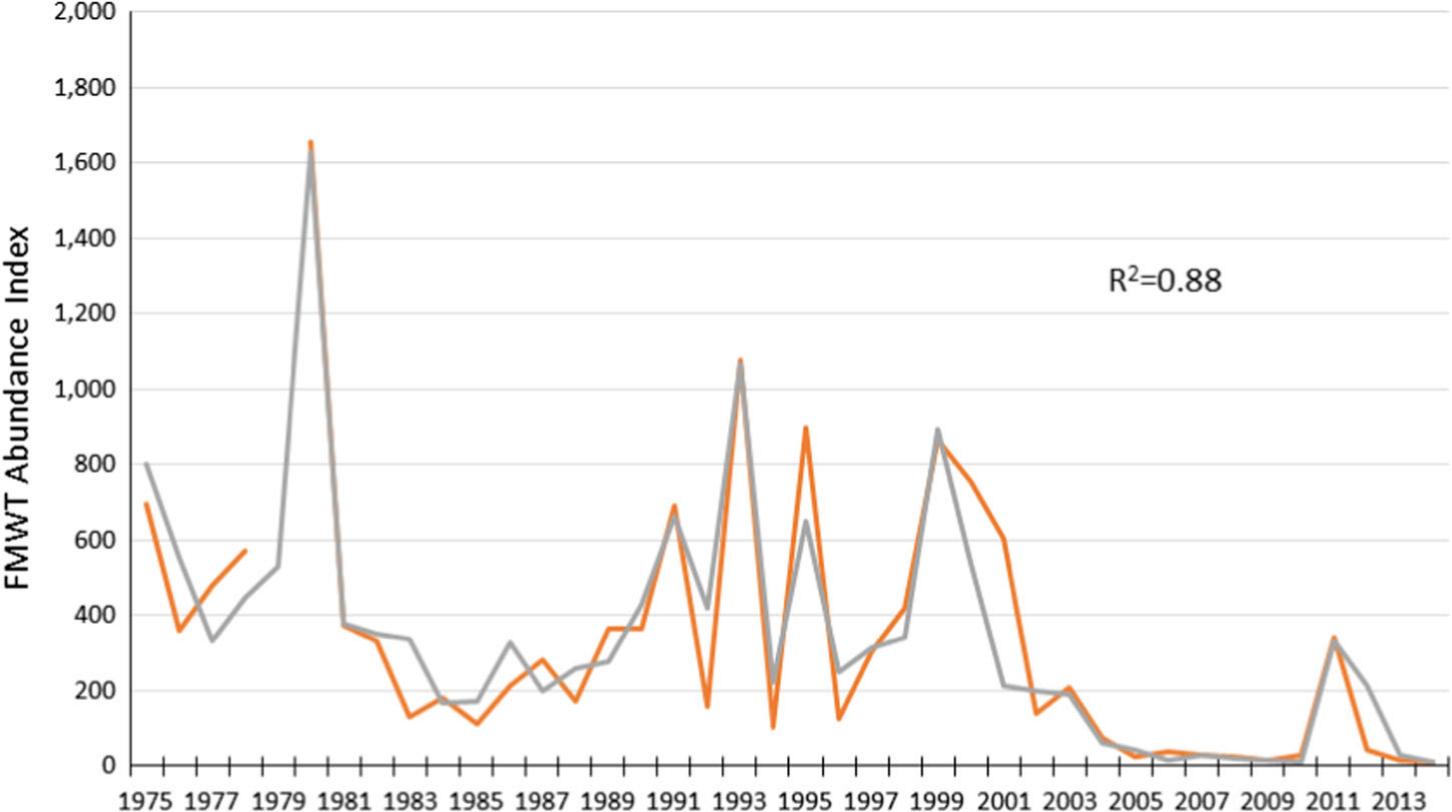
Fall mid-water Trawl Index for delta smelt (orange line) compared to estimates from the model with the lowest AICc value (gray line)

The model generally explained the major changes in the FMWT index (Fig. 3) over four decades (*R*^2^ = 0.88), although it did not explain the timing or the magnitude of the change in several years.

Results from the model with the lowest AIC value indicated that different factors controlled the population in different temporal periods across the time series (Fig. 6, Appendix B). Food has frequently been identified as limiting for delta smelt (for example IEP MAST 2015). Our model identified food limitations in late summer in 17 of 40 years (43%). The model results suggested that food availability in the mid to late 1970s, and again in the 1990s, regulated the population. In other years prior to 1994, losses of delta smelt at power plants appeared to be the primary regulator of population size. Our model suggested that since 2003, abundance of silversides was a primary regulator.

## Discussion

Quantifying the effects of anthropogenic influences on fish populations has been an elusive, unsatisfying, and frequently a controversial endeavor (Rose 2000). Survey-data-based approaches, for the most part, have not provided reliable means of assessing causal relations between environmental factors and fish performance (Rose et al. 2013a). Accordingly, previous studies of delta smelt that have employed additive models have not resolved the causes of its decline (see Mac Nally et al. 2010; Thomson et al. 2010; Maunder and Deriso 2011; Miller et al. 2012).

Among the shortcomings of additive models is that they cannot accurately detect the impacts of factors that limit population performance periodically, intermittently, or infrequently. The relevance of factors that control population responses may be undetected in additive models, leaving the roles of key environmental factors poorly explained. The biological mechanisms implied in additive models are not consistent with the law of limiting factors. Additive models assume that a change in an environmental factor will always contribute to a population response even when that factor does not limit the population. That implicit assumption can lead resource managers and policy makers to incorrectly conclude that benefits to a targeted species from a conservation action are likely when they may in fact not be. Incorporating limiting factors into quantitative analyses has been difficult historically, primarily because environmental factors that regulate a population may manifest inconsistently; an individual factor may control abundance in some seasons and some years and not others.

We combined a conceptual ecological model, supported by ecological theory and a synthesis of the current science (IEP MAST 2015) with mathematical programming that employed a powerful non-linear optimization routine. We applied them in a framework that analyzed the impacts of environmental factors on four delta smelt life stages over 40 years to identify and quantify the roles of those factors in limiting its recovery. We conclude that four of 12 environmental factors explained annual variation in the FMWT abundance index of delta smelt. Entrainment at two power plants had greater effects toward the beginning of the four-decade time period. Predation and competition by an invasive fish, Mississippi silversides, had greater effects in recent years. Food availability in summer appeared to limit delta smelt abundance in more than 40% of the years that were evaluated. Also, the availability of food earlier in the year may influence survival of early life stages. When spring food availability was included in the model, the frequency of food limitation in summer decreased and effects of food limitation in spring were observed.

Although this effort implicates a small set of factors as the primary determinants in the decline of delta smelt, particularly since 2002, the effort to resolve the relative importance of the causes of decline of delta smelt is not yet complete. For example, we did not include contaminants among our covariates due to the lack of adequate data. Also worthy of consideration is predation on delta smelt and its competition with a broader array of introduced predator species (particularly centrarchids), the species richness and abundances of which have increased dramatically over the past three decades. As such, our contribution here does not fully resolve the controversy over the causes of the decline of delta smelt (IEP MAST 2015), but provides a framework with which the causes of the of the decline in delta smelt abundance can be explored as new data become available.

Given the goodness of model fit (Fig. 3), it is fair to ask whether our life-cycle model is “over specified.” Although the model included four covariates, it required the estimation of 10 coefficients. Concerned that models that are over specified may explain ecological phenomena well, but predict them poorly, we carried out a cross-validation analysis, which indicated the model presented here had a relatively robust ability to predict delta smelt responses outside the range of years used to derive the coefficients. Also, rather than being flexible in their functional form, like a third-order polynomial, the logistic functions we employed required that the functions be some form of an “s” curve, further reducing the chance of over-specification. As described earlier, a limiting-factor model subdivides the data, greatly reducing the degrees of freedom and goodness of fit is likely inflated. An area warranting further attention is the development of model selection criteria that discount limiting factors more heavily than modifying factors. Finally, there is the question of whether fluctuations in abundance of delta smelt over four decades in a rapidly changing ecosystem could be adequately explained with yet fewer variables.

The use of non-linear rather than linear models requires estimation of initial values of the coefficients. Use of inaccurate initial values and inclusion of a high number of covariates might lead to the identification of local, rather than global, optima. Therefore, consideration of a range of initial values is required when using this approach. We note that development of a stochastic rather than a deterministic model might reduce uncertainties.

Although we identified 10 geographic regions distributed from the eastern limits of San Francisco Bay to the upper freshwater reaches of the Delta on the basis of landscape and ecological differences, we only had sufficient environmental data for seven of them. The exclusion of part of the range of delta smelt introduces unknown uncertainties into the modeling effort. Furthermore, the use of abundance-index data from the FWMT assumes those data are well correlated with the true size of the delta smelt population; it is not known whether this assumption is valid. In addition, exclusion of a factor from the final model does not mean it has no effect on the species. For example, food availability in spring is essential. We found that food availability in summer frequently controls the population. In the same years that summer food availability is controlling, food availability in spring also can be insufficient, and if food shortages in the summer were to be alleviated, we could expect food availability in spring to control.

The failure to apply the concept of limiting factors has resulted in efforts to increase delta smelt survival or recruitment during individual seasons, rather than to develop a strategy for increasing and sustaining delta smelt abundance across years. For example, the FMWT Index in 2011 was 12 times higher than it was in 2010–a record annual increase in the index. But by 2012, the abundance index returned to near the record low. The benefits of all prior conservation efforts were essentially eliminated by a controlling factor. Rather than focusing conservation efforts toward increasing delta smelt abundance in each individual season, we suggest developing a broader strategy to restore or enhance the resources that limit the recovery of the species. Heretofore, it had been difficult to identify and quantify the impact of limiting factors. Utilizing the tools provided here to identify the resources that likely will be limiting in the future, and employing management actions to relax those limitations, will lead to a more effective allocation of conservation resources and increase population resilience.

### Electronic supplementary material

Appendix B and C

## Figures and Tables

**Fig. 4:**
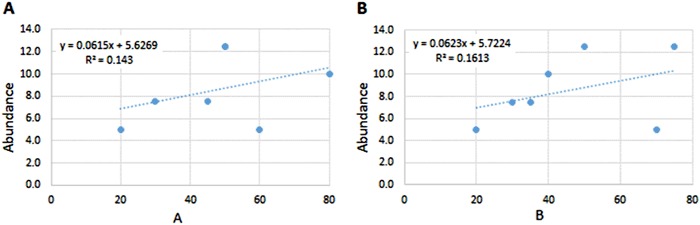
Conventional estimates of relations between simulated covariates **a** (left) and **b** (right) with abundance (the response variable *Y*)

**Fig. 5:**
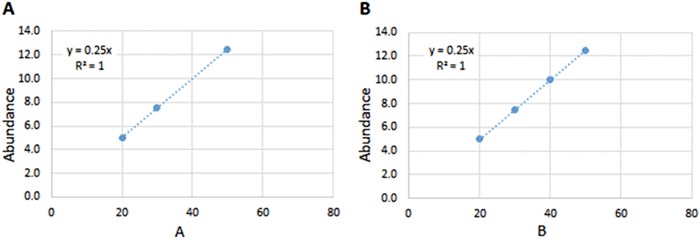
True relations between synthetic covariates **a** (left) and **b** (right) with abundance (the response variable *Y*)

## Appendix A: The Failure of Conventional Correlation Analysis

Not every environmental factor is important in every year, in every season, or in every region. We illustrate the problem with synthetic data (Table 8). Suppose that food availability in summer (covariate *A*) and autumn (covariate *B*) determine the abundance (*Y*) of a species in a particular area. A standard approach might be to hypothesize that one or the other factor is regulating abundance (Fig. 4). Both factors are weakly correlated with the response variable, leading perhaps to a conclusion that neither factor is significant.

**Table 8:** Synthetic data on response variable *Y* given data on covariates *A* and *B*

Year	*Y*	*A*	*B*
	Abundance	Summer food	Autumn food
1	10.0	80	40
2	12.5	50	75
3	7.5	30	35
4	12.5	50	50
5	5.0	60	20
6	5.0	20	70
7	7.5	45	30

Another hypothesis is that both factors are relevant. The results of a multiple regression analysis (Table 9) indicate that neither variable has great statistical significance (*p* values are close to 0.2) although the *R*^2^ of the multiple regression is higher than those of single-variable regressions.

**Table 9:** Results of the multiple regression analysis applied to synthetic data

*R* ^2^	Adj *R*^2^	Standard error	Observations
0.475	0.212	2.823	7

	Coefficients	Standard error	*t* Statistic	*P*-value
Intercept	−0.480	4.885	−0.098	0.926
*A*	0.098	0.0632	1.545	0.197
*B*	0.096	0.0603	1.590	0.187

An apparent anomaly here is the data point (*A*,*Y*) of (60,5) and the next closest *A* observation (50,12.5). If 50 units of *A* yield an abundance of 12.5, how can 60 units of *A* produce an abundance of 5? Dunham et al. (2002) and Cade and Noon (2003) suggested that some other factor limited abundance when *A* = 60. If the data point (*A*,*Y*:60,5) has no bearing on the abundance in autumn, then it should not receive any weight in an analysis, yet conventional approaches assign some weight to it to help explain the variation in abundance.

A better fit for the data is provided by including a pair-wise interaction between *A* and *B*, so the fitted equation becomes:

15 $${{Y}} = \beta _0 + \beta _1{{A}} + \beta _2{{B}} + \beta _3{{AB}} + \mathrm{\mu} .$$

The *R*^2^ then increases to 0.89, leading perhaps to a conclusion that the variation has been largely explained and the results can be used to guide management actions. However, the implications for resource management can be misleading. In our example we assumed that covariate *A*, summer food availability, can be a controlling factor in some years, thereby capping the population potential in the summer in those years. The functional form of Eq. (15) suggests that abundance can be increased (new individuals can be added to the population) by increasing food availability in the autumn. As an extreme example, if summer food availability were to be so low as to cause extinction, the functional form of Eq. (15) would nonetheless suggest that abundance could still be increased by increasing food availability in the autumn. The law of limiting factors asserts that providing more of a given resource, when that resource is not limiting, will not benefit the target species. An inappropriate functional form, despite a good fit to the data, can incorrectly suggest otherwise. Since limiting factors are a fundamental phenomenon in ecology, we caution the use of additive models for guiding management actions.

The synthetic data set contains identities. Each unit of abundance requires 4 units of food in the summer and 4 units of food in autumn. Abundance is regulated by the minimum available food across periods. The underlying relations between *A* and *B* and *Y*, in our example, are linear (Fig. 5). The inability to detect the relevance of limiting factors in standard regression analyses may result in concluding that significant covariates are insignificant, incorrectly estimating coefficients, and inability to explain variances in the response variable (White 2004).

### Alternative approaches

There likely are alternative approaches that can be used to detect the signals of limiting factors in population data. Among them may be the use of inverse models (see for example Tarantola 2005), approximate Bayesian computation (see for example Blum and Francois 2010, Csilléry et al. 2010, Sunnåker et al. 2013), modeling with latent variables (see Masowsky et al. 2014) and analyses incorporating a Random Forest approach (Breiman 2001, Louppe 2015), but it is beyond the scope of this study to compare the applicability of these approaches. Bayesian and Forest analyses are preferable approaches because they implicitly recognize that an existing state is the result of discrete prior events. Approaches that provide a good fit to data without capturing the relevant underlying mechanisms are discouraged.
