# Supplementary material for: Analysis of Limiting Factors Across the Life Cycle of Delta Smelt (Hypomesus transpacificus)
Source: Environ Manage. 2018 May 9;62(2):365–82. doi: 10.1007/s00267-018-1014-9 (PMC6060848; doi:10.1007/s00267-018-1014-9)
Supplement: Supplementary file 1 — Appendix B and C [file 267_2018_1014_MOESM1_ESM.pdf]

Appendix B: Model Validation

We considered the validity of the selected model in six ways. First, we conducted a cross-validation. The cross-validated correlation between the predicted, omitted observations and the FMWT Index was 0.90 (R^2=0.82). The coefficients were consistent between time periods with all coefficients having a coefficient of variation of less than 10%, indicating stability in the model, and 8 of the 10 having a coefficient of variation less than 5% (Table 10).

Second, we checked for adequate degrees of freedom. Partitioning the data into two subsets reduces the degrees of freedom and increases the risk of over-specification – that is, having too many coefficients for the available observations. As our rule of thumb, we desired to have degrees of freedom that were double the number of coefficients being estimated. Beginning with equation (1) through (4) we eliminated excluded covariates, determined the number of observations in each subset of the data, and checked for degrees of freedom in each. The selected model included two limiting factors, prior abundance and food availability in summer. Effectively this means the data were divided into two subsets with two separate equations being estimated. We checked the degrees of freedom for each equation.

Eliminating factors not included in the selected model, equations [1] to [4] become

$$A_m = A_f \tag{9}$$

$$A_r = A_m \, M_2 \, f(E_p) \, f(P_s) \tag{10}$$

$$A_j = \text{Min} \{ [A_r], \ [g(F_{JA})] \} \tag{11}$$

$$A_e = A_j \tag{12}$$

When food availability in summer is limiting, we can substitute equation (11) into (12) to give

$$A_e = g(F_{JA}) \tag{13}$$

This equation requires the estimation of 3 coefficients for $g(F_{JA})$. Equation (13) was applied to 17 years of data, providing 13 degrees of freedom.

When food availability is not limiting, we can substitute equation (9) into (10) into (11) into (12) to give

$$A_e = A_f \ M_2 \ f(E_p) \ f(P_s) \tag{14}$$

This equation requires the estimation of 6 coefficients {3 for $f(E_p)$, 3 for $f(P_s)$} and 1 parameter. Equation (14) was applied to 22 years of data leaving 14 degrees of freedom. In both cases the number of degrees of freedom met our criterion of being twice the number of parameters being estimated.

Third, we considered other data to assess the plausibility of results. Power-plant operations were a potential concern because of the effects of entrainment, and both temperature and toxicity of discharged water (Matica and Sommer 2005). The two plants combined at maximum capacity could circulate 3,240 cfs or 10,500 acre feet of water per day (Matica and Sommer 2005). Entrainment of larval smelt (both delta smelt and longfin smelt) during one year (March 1978-March 1979) were estimated to be 20.5 million (+/- 5.6 million) at the Contra Costa Power Plant and 64.7 million (+/- 29.5 million) at the Pittsburg Power Plant for a total of 85 million smelt larvae (Matica and Sommer 2005). Due to difficulties in identifying young smelt, it was not possible to differentiate delta smelt larvae from longfin smelt larvae. For older smelt that could be identified, 100% and 70%, were identified as delta smelt at Contra Costa Power Plant and Pittsburg Power Plant, respectively. Collectively, these data suggest that power plant operations may have regulated the population in the past. The model results suggest that power plant operations

Table 10 Coefficients of variation from the cross-validation analysis

	β_0	β_1	β_4	
Food Avail. (Jul-Aug)	-2.5%	6.0%	9.6%	
Silversides Abundance	0.7%	-1.4%	2.6%	
Power Plant Operations	4.2%	-1.7%	3.4%	
Recruitment Parameter				3.0%

have not regulated the delta smelt population since 1993.

The abundance of predaceous Mississippi silversides in the estuary (see Figure 6c) has increased considerably; the species has displaced native species in other areas in the United States (USGS Fact Sheet https://nas.er.usgs.gov/queries/FactSheet.aspx ?SpeciesID=2903); delta smelt DNA was previously detected in 12.5% of silversides (Schreier et al. 2016) with a method that could detect delta smelt DNA up to 36 hours post ingestion (Baerwald et al. 2012), and the distribution of silversides appears to have shifted to the western Delta (Mahardja et al. 2016), an area in which densities of delta smelt at certain times of the year are relatively high (Merz et al. 2011). Given that silversides are one of the more abundant non-native piscivores in the Delta and adjacent bay and marsh complex, it seems ecologically plausible that silversides may have a regulating effect on the species (Schreier et al. 2016). Although the model results suggest that silversides might affect the delta smelt population via predation during spring, given the negative correlation between abundance of silversides food availability in July and August of -0.56, the effect might also be manifested through competition for food.

Fourth, we considered the plausibility of the shape of the response functions. Using the estimated coefficients from the model with the lowest AICc value, we graphed the estimated response functions (see equation (5a), (5b) and (5c)) for food availability (Figure 7a), power plant operations (Figure 7b) and abundance of silversides (Figure 7c). Our results suggest that food availability in July and August can be limiting over wide areas of the Delta, but that densities of 4,000 gC/m^3 are necessary to sustain a FMWT index value of around 1,000. The shape of the response function for power plant operations seemed plausible with survival dropping off sharply at power generation levels above 500 MWH. The response function for silversides, however, did not seem ecologically plausible. Survey data (including STN survey data that were not utilized in the model) would lead one to expect a gradual s-shaped function, but instead the response curve declined rapidly above silversides abundances of 120 (see Figure 7c).

Fifth, we correlated our predicted delta smelt seasonal abundances with survey returns from the Spring Kodiak Trawl (average February CPUE), the 20mm Survey (average June CPUE), and the Summer Tow-net Survey (average July CPUE) to see if the factors affecting abundance were incorporated in the correct life stages, recognizing that our life stages do not correspond exactly with survey data (Table 11). Correlations between the model and survey data generally were equal to or higher than correlations with the prior FMWT index.

Sixth, we evaluated whether the timing of modeled life-stage impacts were consistent with the survey data. If the delta smelt abundance index value in one season is a good

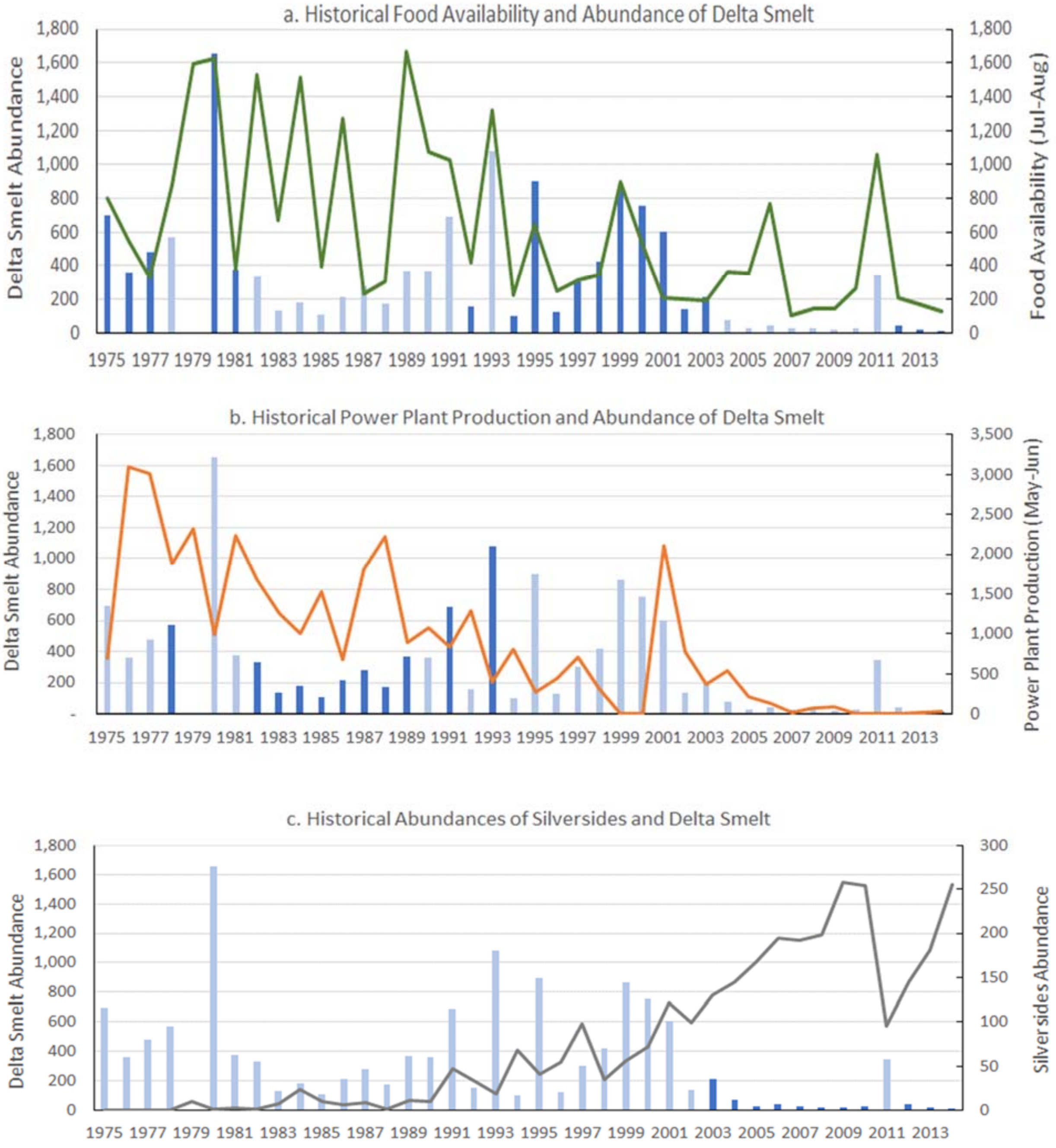

Fig. 6 Historical delta smelt abundance-index values (Fall Mid-water Trawl Index, blue columns) and covariates that model outputs suggested may regulate the population: a. Food Availability Jul-Aug, b. Power Plant operations, and c. Abundance of silversides (predation). Dark blue columns designate years in which the model indicates that each respective factor regulated abundance.

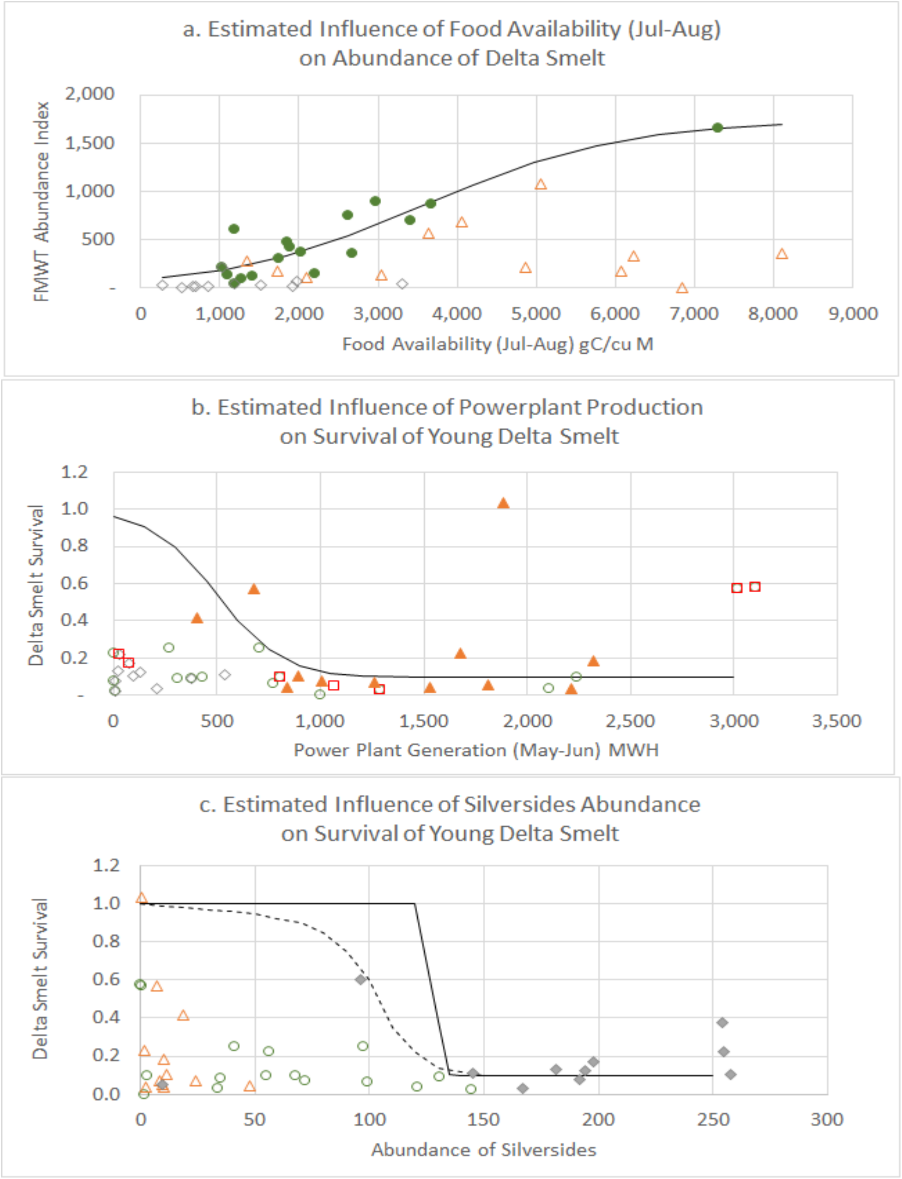

Fig. 7 Estimated associations between covariates and abundance or survival of delta smelt. Circles denote years when food availability in July and August was limiting, triangles denote years when energy generation at power plants was the primary factor regulating the population, and diamonds represent years when silversides were the primary factor regulating the population. Black solid lines represent response curves fitted by the model. (a) Relationship between food availability in July and August (from the zooplankton survey) and delta smelt abundance (from the FWMT Index). (b) Relation between power plant generation and survival of young delta smelt (as calculated by a ratio of the STN index/prior FMWT Index, scaled to fit the y axis). Red squares reflect drought years when delta smelt were likely not near the power plants. (c) Relation between silverside abundance and survival of young delta smelt.

Table 11 Correlations of model estimates with metrics of abundance of delta smelt at each life-stage and with the prior FMWT Index.

	Pre-spawning adults	Recruits	Sub-juveniles	Sub-adults
Metric	SKT CPUE Feb	20 mm CPUE Jun	STN Index	FMWT Index
Period	2002-2014	1995-2014	1995-2014	1995-2014
Prior FMWT Index	0.92	0.71	0.76	0.43
Model Estimate	0.92	0.87	0.74	0.92

predictor of the index value in the next season, environmental factors likely have had little effect on population during that period. When the between-season variation is substantial it indicates that environmental factors have had comparatively greater influence in regulating the population during that period. A plausible model should include environmental factors in those life stages for which abundance metrics are not well correlated with abundance metrics in the previous life stage, and should not include factors where the abundance metrics are well correlated with abundance metrics of previous life stages. We identified life-stage transitions that likely were most strongly influenced by environmental factors (Table 8). There appeared to be relatively little regulation of abundance by environmental factors in transitions from autumn to winter and from spring to summer. The model did not include covariates from these transitions. Winter to spring (adults to recruits) was more strongly regulated by environmental factors. The model with the lowest AICc value included two factors from this transition: energy generation by power plants and silversides abundance. The greatest variation in regulation by environmental factors was from summer to autumn. The model with the lowest AICc value included the limiting factor of food availability Jul-Aug. Based on the foregoing analyses, we concluded that the covariate stressors were generally associated with appropriate life stages.

We concluded that the model with the lowest AICc value was statistically rigorous and explained much of the variation in the annual abundance of delta smelt over four decades. The covariates that were included were plausible ecologically, appeared to be associated with appropriate life stages and the (potential) magnitude of their impact on delta smelt abundance was supported by field data. The shape of the response functions was plausible for two of three covariates, but the response function for silversides included a threshold that may be implausibly abrupt. The inclusion of a model containing food availability in the spring, while not as statistically strong, seems more plausible ecologically.

References

Baerwald MR, Schreier BM, Schumer G, May B (2012) Detection of threatened delta smelt in the gut contents of the invasive Mississippi silverside in the San Francisco Estuary using TaqMan Assays. Trans Am Fish Soc 141:1600–1607

Mahardja, B, Conrad J, Lusher L, Schreier, B (2016) Abundance, trends, distribution and habitat associations of the invasive Mississippi silverside (*Medindia audens*) in the Sacramento-San Joaquin Delta, California, USA. San Francisco Estuary Watershed Sci 14(1):1–16

Matica Z, Sommer T (2005) Aquatic impacts of the Pittsburg and Contra Costa Power Plants, Draft Report. CDWR

Merz JE, Hamilton SA, Bergman PS, Cavallo B (2011) Spatial perspective for delta smelt: a summary of contemporary survey data. California Fish and Game 97:164-189

Schreier BM, Baerwald MR, Conrad JL, Schumer G, May B. (2016) Examination of Predation on Early Life Stage Delta Smelt in the San Francisco Estuary Using DNA Diet Analysis. Trans Am Fish Soc 145:723-733

Appendix C: Procedure Used to Conduct an Analysis to Identify Limiting Factors

In developing a procedure to conduct an analysis to identify limiting factors for delta smelt, we drew from Rose et al. (2015) and Swannack et al. (2012). Appendix A of Rose et al. (2015) provides detail on each of their steps. Note that several of the steps are associated with the development of a restoration plan in a public review process. Recognizing that these steps are important and appropriate, but beyond the scope of the work here, they have not been included below.
The information in italics below briefly describes our implementation of each of the steps we employed – a synthesis from Rose et al. (2015) and Swannack et al. (2012). Reference to the Manuscript refers to: Hamilton and Murphy (2018). Reference to a table or a figure with the prefix LFM is to an Excel file: LFM-no links.xlsx which we have made available at:

https://www.dropbox.com/s/vt4qkq52t66e8ib/LFM%20-%20no%20links.xlsx?dl=0

Step

1. Articulate the problem, objectives, and questions to be answered.

   Given that the "results of (prior) quantitative analyses of multiple environmental stressors on delta smelt have been inconsistent" our objective was "to explain variation and trend in a common abundance index for of delta smelt" (from the introduction of the manuscript) in order to aid the development of an effective management strategy that could provide sustained benefits to delta smelt.

2. Review pertinent theory and literature, historical circumstances and previous studies.

   This was included in the manuscript as appropriate. Also see manuscript references.

3. Create and unify conceptual ecological models. Summarize prior knowledge.

   We reviewed previous conceptual models but did not include that review in the manuscript. We then adapted the model of Moyle et al (2016). See: Figure LFM-2. See also Manuscript: Figure 2 and sections titled "Conceptual Ecological Model" and "Candidate Covariates and Model Structure".

4. From the conceptual model, and specifically the transition stages within the conceptual model, build a library of candidate quantitative models:

   a. *We identified the transition stage for which the most reliable survey data exists to establish stock, selecting the Fall Mid-water Trawl Index. In the Manuscript, refer to the third paragraph under conceptual ecological model for the basis of selection.*

   b. *We worked through each transition stage of the life cycle, identifying mechanistic factors affecting recruitment or survival, as relevant. In the Manuscript, see equations (1), (2), (3) and (4).*

   c. *We transformed the factors into covariates.*

      i. Create a data inventory, documenting all the data.
      This was done in the workbook LFM.xlsx and preceding workbooks. The data used in the analysis is presented in Table

LFM-14a. The data sources are documented in Table 1 in the Manuscript. The derivation of these covariates is provided in Table 1 of the Manuscript. The food availability covariates were calculated as follows:

- *The average biomass in each region in each month of each year was calculated using zooplankton survey data. See: e.g. Table LFM-3a. To calculate biomass, we used the grams of carbon for each species listed in footnote [a] of Table 1 of the Manuscript.*
- *To reflect access to prey (food) by region, we calculated for each region and month, the percentage of stations with abiotic factors, as recorded in the zooplankton survey, that fell within the attribute ranges listed in Table 2 of the Manuscript. See e.g. Table LFM-3b.*
- *We calculated, for each region and month, what we call a "habitat suitability score," which is the product of the average biomass and the percentage of stations with suitable conditions. See e.g. Table LFM-3c.*
- *Then we averaged scores across regions to calculate what we call a "capacity score" which is the value of the covariate used in the analysis. See the right-hand column in Table 3c.*
- *This procedure was used for all months except spring (April-June), where the average was calculated only for Suisun Marsh, the Confluence and Lower Rivers regions, and the prey range was extended to include smaller prey items: calanoid copepodids and cyclopoid adults.*

ii. If data are not available for a factor, consider providing reasonable proxy variables or reasonable estimates of the factor.
In the Manuscript, see narrative preceding each of equations (1)-(4)

d. Graph the covariates against abundance or survival, where possible.

See: Figure LFM-3, Graphs off individual covariates vs Abundance or Survival

e. Check the covariates for correlation.

See: Table LFM-14a Correlation among candidate covariates (Manuscript Table 3)

Select the statistically most relevant food covariate to reduce the influence of correlations in the analysis. See Table LFM-14a (Table 4 in the Manuscript). Noted correlations when interpreting results.

f. Articulate the hypothesized relationships between the covariates and abundance at each life stage as equations.

We distinguished the factors that might limit the population potential versus factors that operate after a population potential been established (modifying factors). Our guidelines:

- *Prior population is a limiting factor. Food availability is a limiting factor – that is, if insufficient food is available, the population cannot be maintained. Habitat space could be a limiting factor, but with abundance indexes at record lows it is unlikely to be constraining abundance. Habitat space may be implicitly considered as diminishing access to food.*
- *Anything that kills a portion of the population (as opposed to stressing*

the whole population) is a modifying factor, e.g. entrainment, disease, or predation.

- *Abiotic environmental attributes are most likely modifying factors. Water temperature, if sufficiently high, could kill the entire population; however, due to occupancy by delta smelt of diverse and varying habitat niches and the wide variation in water temperature manifested across the estuary, the entire delta smelt population would not likely be simultaneously directly affected by elevated temperature. Should water temperature determine the extent of habitat then it could be a limiting factor.*

The hypothesized relationships are that each of the factors listed in Table 1 of the Manuscript has a strong statistical association with the FWMT Index, therefore can help explain variations in it. We recognize that informatic theoretic analyses cannot be used to truly "test" hypotheses, but rather they provide inferences.

5. Computerize the quantitative model:

   a. Identify, import, and check the data.
   See: Table LFM-14a Candidate Covariates.

   b. Incorporate the equations for each life stage into the quantitative model.
   See: Table LFM-15 Calculation of estimated FMWT Index and RSS.

   c. Set up the statistical calculations: RSS, TSS, R^2, adj R^2, AICc etc.
   See: Table LFM-15b Summary Statistics.

   d. Write up a draft of the methods to help catch logic errors.
   In the Manuscript, see "Methods".

   e. Verify the model.

   Checked formulas and data.
   In order to confirm whether Excel's nonlinear optimization routine was sufficiently precise, we checked a subset of the results against results using R. In the preferred model, no modifying factors alter the estimated abundance index values resulting from food availability limitations in Jul-Aug. By extracting the data for the 17 years in which Food Availability Jul-Aug was determined to be limiting, we could independently estimate the coefficients for equation [5c] that are reported on line [19] of the worksheet "Model." We found no difference in the estimated coefficients between R and Excel to 3 decimal places.

6. Run the analysis

   a. Develop reasonable starting values for the coefficients.
   These are inserted into the colored cells in Table LFM-15a.

   b. Run the analysis using non-linear optimization to minimize RSS.
   We activated the "solver" add-in in Excel. The objective function to be minimized is the RSS: cell I34 in worksheet model. We identified the cells to be changed - the coefficients to be estimated. In this version of the model these cells are shaded blue. It can be useful to add constraints, especially on minimal survival. These constraints may not actually constrain the coefficients; however, specifying those constraints prevents the optimization routine from producing a solution with negative survival. We uncheck the box below the constraints box labeled: Make unconstrained variables non-negative.

Under options, in the GRG Tab, we changed the default convergence precision from 0.0001 to 0.0000001.
We activated the solution routine by clicking the solve button. If a feasible solution was not found, we modified the starting values.

c. Graph the results.
See: Figure LFM-7 Time Series of Estimated Versus Actual FMWT Abundance, Figure LFM-8 Scatter Diagram of Estimated Versus Actual FMWT Abundance.

d. Check the results for reasonableness and obvious errors (e.g. calculation errors).
This was done at the end of each model run, and again at the end of all runs.

e. Check for global optimality.
We re-ran models using different starting values to assess the stability of the solutions, and establish whether a lower AICc value could be found.

f. If different functional forms (log, natural, quadratic, etc) are reasonably defensible, run the alternatives and see what provides the best fit, but do not let the best fit override theory.
While simple linear models, rather than logistic functions [Manuscript equations (5a), (5b), (5c)], might be more efficient statistically for some life stages (that is, they can produce lower AICc values), for consistency and to reduce data mining, we maintained consistent functional forms.

g. Sequentially omit covariates to see if any do not provide valuable information, as determined using the model-selection criteria.
See: Worksheet "Results" (Tables 4 and 5 of the Manuscript).

h. Preserve the results of each run.
See: Table LFM-16 Analysis of Contribution of Individual factors, Table LFM-17 Consequences of adding or deleting covariates to the preferred model, Table LFM-18 Consequences of adding or deleting covariates to the March food model.

i. Develop a set of best models using conventional statistical techniques.
The selected model (the model with the lowest AICc) was model 30 in Manuscript Table 5 and was the base model in Table 6. A model that also included food availability in March had the next lowest AICc value (2.9 higher than the selected model). (See Manuscript Table 6).

7. Validate the best models.
See Appendix B.

a. Perform cross validation or other analyses.
See: Table LFM-22b Cross-validation output.

b. Check reality of results against data - are coefficients realistic?
See: Figure LFM-9 Estimated associations between covariates and abundance or survival of delta smelt, Table LFM-24e Summary of Correlation between Model Estimates & Survey Results,Figure LFM-11 Influence of the abundance of delta smelt in one life stage on abundance in a subsequent life stage from survey data.

8. Perform sensitivity and uncertainty analyses.

We checked model sensitivity by including and excluding covariates into or from the selected model (See Manuscript Table 6).
We checked to see if models including food availability in both March and Jul-Aug could improve results. See Table LFM-18.

9. Select a preferred model based on the purpose (management relevance of the model), statistical strength, and ecological credibility.

   After reviewing the selected model from both an ecological and statistical perspective (see Appendix B), we considered Model 30 to be suitable for the purposes of this study.

References

Hamilton SA, Murphy D.D. (2018) Analysis of limiting factors across the life cycle of delta smelt (*Hypomesus transpacificus*). Environmental Management

Moyle PB, Brown LR, Durand JR, Hobbs JA (2016) Delta smelt: life history and decline of a once-abundant species in the San Francisco Estuary. San Francisco Estuary Watershed Science 14:1–30

Rose KA, Sable S, DeAngelis, DL, Yurek, S Trexler JC, Graf, W Reed DJ (2015) Proposed best modeling practices for assessing the effects of ecosystem restoration on fish. Ecological Modelling 300:12-29

Swannack TM, Fischenich JC, Tazik, DJ. (2012) Ecological Modeling Guide for Ecosystem Restoration and Management. U.S. Army Corps of Engineers, EDRC/EL TR-12-18, Vicksburg, MS
